# PLNC8 αβ Potently Inhibits the Flavivirus Kunjin and Modulates Inflammatory and Intracellular Signaling Responses of Alveolar Epithelial Cells

**DOI:** 10.3390/v16111770

**Published:** 2024-11-13

**Authors:** Abubakr A. M. Omer, Sanjiv Kumar, Bo Söderquist, Wessam Melik, Torbjörn Bengtsson, Hazem Khalaf

**Affiliations:** School of Medical Sciences, Faculty of Medicine and Health, Örebro University, 701 82 Örebro, Sweden; abubakr.omer@oru.se (A.A.M.O.); drsanjivk@gmail.com (S.K.); bo.soderquist@oru.se (B.S.); wessam.melik@oru.se (W.M.); torbjorn.bengtsson@oru.se (T.B.)

**Keywords:** PLNC8 αβ, antiviral, flavivirus, inflammation, MD simulation

## Abstract

PLNC8 αβ is a cationic antimicrobial peptide that previously has been reported to express both antibacterial and antiviral properties. This study aimed to further elucidate the antiviral effects of PLNC8 αβ and its impact on virus-induced cytotoxicity and inflammatory signaling in human alveolar epithelial cells (A549) infected with the flavivirus Kunjin. Complementary in silico analyses using molecular dynamics (MD) simulation were conducted to investigate the mechanism of action of PLNC8 αβ by studying the interaction of PLNC8 α and β with models of a flavivirus membrane and a eukaryotic plasma membrane, respectively. Our findings demonstrated that PLNC8 αβ significantly reduces both extracellular and intracellular viral loads, as confirmed by plaque reduction assays and RT-PCR. The peptide also mitigated virus-induced cytotoxicity and inflammation. Notably, PLNC8 αβ modulated the virus-induced dysregulation of key signaling and inflammatory genes, such as *TLR9*, *TLR3*, *NOD2*, *FOS*, *JUN*, *IL6*, and *CXCL8*. MD simulation revealed that PLNC8 αβ exhibits higher binding affinity for a flavivirus membrane model compared to a model of the plasma membrane, likely due to stronger electrostatic interactions with anionic phospholipids. This selective interaction possibly accounts for a potent antiviral activity of PLNC8 αβ combined with a minimal cytotoxicity toward human cells. Overall, PLNC8 αβ shows significant promise as an antiviral agent against flavivirus infections and warrants further exploration for peptide-based antiviral therapies.

## 1. Introduction

Antimicrobial peptides (AMPs) are a diverse group of molecules produced by virtually all living organisms as part of their innate immune system [1]. Research into the potential therapeutic roles of AMP during the last decade has increased with postulated antibacterial, antiviral, antifungal and even anticancer effects in multicellular organisms [2,3,4]. The mechanisms of action (MOA) of most AMPs are not fully elucidated but it is suggested to vary depending on the type of peptide and the target microorganism [5,6]. Both direct and indirect MOA are suggested for AMPs. The direct MOA includes binding to microbial membranes, causing their disruption, or targeting of intracellular components and processes [7,8,9]. The former mechanism is widely accepted for cationic AMPs, involving electrostatic interaction with negatively charged phospholipids, ultimately leading to membrane disruption [10,11,12]. The indirect MOA of AMPs is suggested to consist of modulation of the inflammatory and immune responses of the host [9,13,14].

AMPs exert their immunomodulatory activity in various pathological conditions by affecting key inflammatory signaling pathways, such as Toll-like receptor (TLR) signaling through the nuclear factor kappa B (NF-κB) pathway and the mitogen-activated protein kinase (MAPK) pathway [15,16]. TLRs and other pattern recognition receptors (PRRs), such as nucleotide oligomerization domain (NOD)-like receptors (NLRs) and retinoic acid-inducible gene-I (RIG-I)-like receptors (RLRs), are essential parts of the innate immune response against virus infections by sensing nucleic acids and other viral components. This leads to interferon expression and activation of other pathways involved in protection against viral infections [17]. Interestingly, TLR3 has been shown to play an important role in protection against the flavivirus West Nile Virus (WNV), since mice deficient in TLR3 are more vulnerable to severe forms of encephalitis caused by this virus [18]. Furthermore, another study demonstrated the role of the downstream signaling molecule MyD88 in restricting the replication of WNV and influencing the expression of chemokines [19]. MyD88 is one of the canonical adaptors that are crucial for downstream signaling of TLRs and other PRRs [20]. AMPs are thought to modulate TLR signaling pathways through different mechanisms, for example by forming complexes with natural TLR ligands, such as ssRNA and dsRNA, enabling them to get access to the endosomal TLRs of immune cells with subsequent activation of downstream inflammatory pathways [21]. The NF-κB and MAPK signaling pathways are among the key cellular signaling pathways that are activated in response to infections by various types of DNA and RNA viruses, as shown in studies involving many types of human and animal cells [22,23]. NF-κB is a family of highly conserved transcription factors that play essential roles in cellular inflammatory and immune responses [24]. In addition to TLR signaling, the NF-κB pathway is induced by other immunological stimuli that includes members of the Tumor Necrosis Factor (TNF) family of cytokines and IL-1β [25,26]. Important members of the MAPK family of transcription factors include the extracellular signal-regulated kinase (ERK), p38, and c-Jun NH (2)-terminal kinase (JNK), and several studies have elaborated on their roles in orchestrating the cellular response in various pathological conditions [27,28].

AMPs have been suggested to induce anti-inflammatory effects by inhibiting key intracellular signaling pathways [29,30] and to affect the expression and secretion of chemokines and other inflammatory mediators. For example, defensin, which is naturally secreted by stimulated neutrophils, has been shown in vitro to induce the expression of CXCL8 by the airway epithelial cells A549, thereby further enhancing the recruitment of neutrophils [31]. The human AMP LL-37 has also been shown to upregulate the expression of CXCL8 by A549 cells [32].

WNV is a flavivirus that is found in virtually all continents causing frequent emerging public health threats [33]. WNV is phylogenetically related to the group of medically relevant vector-borne flaviviruses, such as yellow fever (YFV), dengue (DENV), Zika (ZIKV), Tick-borne encephalitis (TBE), and Japanese encephalitis (JEV) [34]. Kunjin (KUNV) is a member of the WNV lineage 1, with close relevance to other WNVs in terms of ecology, epidemiology, and cross reactivity [35,36]. Although there are effective vaccines for some flaviviruses, such as YFV, TBE, and JEV, there are no licensed drugs for most flaviviruses, including DENV, WNV and KUNV [37,38].

Plantaricin NC8 αβ is a bacteriocin derived from the bacterium *Lactobacillus plantarum*. PLNC8 αβ is composed of two peptides, α and β, that work synergistically to exert their antimicrobial activity. The primary mechanism of action is presumed to be through high affinity electrostatic interactions with negatively charged bacterial membrane structures, such as phosphatidylglycerol, leading to membrane disruption and bacterial cell death [39,40]. Interestingly, we have previously shown that PLNC8 αβ also possesses significant antiviral activity against several enveloped viruses. Notably, the peptides are particularly effective against viruses that obtain their envelopes from the endoplasmic reticulum (ER), such as flaviviruses and SARS-CoV-2 [41]. The main objective of this study is to further characterize the antiviral activity of PLNC8 αβ on KUNV-infected cells and to explore their subsequent effects on host cell responses by studying intracellular signaling events, generation of inflammatory mediators, and their ability to mitigate virus-induced cytotoxicity. Additionally, by using complementary in silico methods, we aimed to study the interaction of PLNC8 α and β with models of a viral membrane and the plasma membrane, respectively, to gain deeper insights into the mechanism behind the antiviral activity of PLNC8 αβ and their low cytotoxicity to human cells. Clarifying these aspects of the antiviral properties and MOA of PLNC8 αβ, along with its potential modulatory effects, is crucial for the development of a new class of potent antiviral compounds.

## 2. Materials and Methods

### 2.1. Cell Culture

Human lung carcinoma epithelial cells (A549, ATCC CCL-185) were used for the infection experiments and the monkey kidney epithelial cells (Vero, ATCC, CRL-1586) were utilized for determining the extracellular viral count using plaque assay. Both cell lines were purchased from the American Type Culture Collection (ATCC) and maintained in Dulbecco’s Modified Eagle’s Medium (DMEM) containing 1 g/L glucose (Gibco, Paisley, UK), supplemented with 10% heat-inactivated fetal bovine serum (HI–FBS, Gibco) and 100 U/mL penicillin-streptomycin (PEST, Gibco) and incubated at 37 °C in 5% CO_2_.

### 2.2. Virus Strains and Propagation

Stocks of Kunjin (KUNV) were prepared in Vero cells. Briefly, the cells were infected by KUNV and then grown in complete DMEM containing 2% HI-FBS at 37 °C in 5% CO_2_ for 3–4 days, when an obvious cytopathic effect (CPE) is usually observed. The cell culture medium was initially centrifuged at 2500× *g* and 4 °C for 5 min to remove the debris and then collected and purified by ultracentrifugation over a 20% sucrose cushion at 150,000× *g* and 4 °C for 2.5 h, using Beckman Coulter’s Optima-XPN ultracentrifuge (Indianapolis, IN, USA). The virus-containing pellet was resuspended in complete DMEM medium (in 1% HEPES buffer), and the virus concentration was quantified by performing plaque assays.

### 2.3. Peptides

The peptides were purchased from GL Biochem (Shanghai Ltd., Shanghai, China). The characteristics of the peptides, including the molecular weight, net charge at pH 7 and the amino acid sequences are shown in Table 1. The *L*- and *D*- in the peptide names (*L*-PLNC8 αβ, *D*-PLNC8 αβ) refer to the configuration of the amino acid residues in the peptide chains. Scrambled versions of PLNC8 α and β (*S*-PLNC8 α, *S*-PLNC8 β), which are composed of the same amino acids as the native peptides but are randomly shuffled in new positions, have been used as a control to verify that the effects of the peptides are specific and dependent on the amino acid sequence of their primary structure.

### 2.4. Viral Infection and Peptide Treatment of A549 Cells

A549 cells were seeded in appropriate cell culture plates and incubated in a stable environment for 24 h to reach a monolayer of confluent cells. The cell culture media was then removed, and the cells were infected with KUNV at a multiplicity of infection (MOI) of 0.1. After 1 h of virus adsorption, the virus inocula were removed and the cells were washed with PBS before adding either *L*-PLNC8 αβ or *D*-PLNC8 αβ at a final concentration of 10 µM diluted in new media. At 48 h post-infection (HPI), cell culture supernatants were collected, centrifuged, and kept at −70 °C until further use to determine the cytotoxicity and the levels of inflammatory mediators. The cells were used for RNA extraction and protein isolation. In another set of experiments, the peptides were added in 3 doses (one dose/day) using three different concentrations (0.1, 1, and 10 µM). The viral load was assessed by performing plaque assay after 72 h. In another experiment, human lung carcinoma cells (A549) were infected with KUNV for 24 h, followed by administration of *L*-PLNC8 αβ (*L*-αβ) or *D*-PLNC8 αβ (*D*-αβ) at 10 µM and 20 µM for 2 h before assessing the viral load using plaque assay. As a control, cells were exposed to heat-inactivated viral particles instead of viable viruses. The viral particles were inactivated by exposing them to 70 °C heating for 20 min.

### 2.5. RT-PCR

After 48 h post infection, total RNA was extracted from cultured cells using E.Z.N.A Total RNA Kit I (OMEGA Bio-Tek. Inc., Norcross, GA, USA) according to the instructions of the manufacturer. cDNA was synthesized using High-Capacity RNA-to-cDNA Kit (Thermo Fisher Scientific, Baltics UAB V.A., Vilnius, Lithuania) according to manufacturer’s instructions. Realtime PCR was performed using Power-Up SYBR Green Master Mix (Applied Biosystems, Waltham, MA, USA). The sequences of all primers used are shown in Table 2. Relative gene expression levels were calculated using the ∆ΔCt method.

### 2.6. Plaque Assay

The antiviral activity of *L*-PLNC8 αβ and *D*-PLNC8 αβ against KUNV was determined by comparing the infectious virus titers, which were measured by crystal violet-based plaque assay. Cell culture supernatants were collected from treated and untreated cells at 48 HPI in the single-dose treatment experiments and at 72 HPI in the multiple-dose experiments. Briefly, a series of dilutions of the supernatants were used to infect a 90% confluent layer of Vero cells for 1 h at 37 °C, followed by covering the cells with overlay media (DMEM supplemented with 1.2% Avicel (FMC, Piladelphia, PA, USA), 2% HI–FBS (Gibco, Paisley, UK), 1X non-essential amino acids (Gibco), and 1% PEST (Gibco)). After 3 days, the overlays were removed, and cells were washed with Phosphate Buffer Saline (PBS) and subsequently fixed with 100% methanol for 30 min. The cells were then stained with 2% crystal violet (Sigma, Saint Louis, MO, USA), 20% methanol (Fisher, Loughborough, Leicestershire, UK), and 0.1% ammonium oxalate (Sigma) solution for 1 h, and then washed with water. Finally, the plates were placed upside down at room temperature overnight and the number of virus plaques counted.

### 2.7. ELISA

Using samples from the cell culture supernatants collected at 48 h post infection, we performed quantitative analysis by ELISA for CXCL8, and IL-6. Commercial kits (Invitrogen™, Bender MedSystems GmbH, Vienna, Austria) were used according to the manufacturer’s instructions and the absorbance was finally quantified in a plate reader set at 450 nm.

### 2.8. Cytotoxicity Assay

Cytotoxicity was determined by assessing the activity of lactate dehydrogenase (LDH) in cell culture supernatants, using LDH Cytotoxicity Assay Kit (Thermo Scientific™) and following the instructions of the manufacturer. Intracellular LDH released by damaged cells catalyzes certain reactions that ultimately change some of the chemicals used in the assay to a colored formazan product which is measured at 490 nm reflecting the amount of LDH.

### 2.9. Western Blot

A549 cells were lysed in RIPA buffer (Millipore, Burlington, MA, USA). Micro BCA™ Protein Assay kit (Thermo Scientific, Rockford, IL, USA) was used for quantification of total protein concentration according to manufacturer’s instructions using the Cytation plate reader, Biotek (540 nm). Final calculations and analysis of the protein concentration were done using the online tool MyAssays. For Western blot analysis, 30 mg of total protein was heat-denatured at 95 °C (in cell lysis buffer mixed with 4× SDS), then separated by 10% SDS–PAGE and transferred to a nitrocellulose membrane for assaying of phosphorylated p65 (Novus Biologicals, NB100-82088, Centennial, CO, USA) and tubulin (Merck Life Science AB, Catalogue number 05-661, Stockholm, Sweden). Finally, ECL detection solution (Millipore, Catalogue number WBKLS0100) was added to the membrane and the densities of the protein bands were determined by using LI-COR Image Studio software, version 3.1 (Biosciences, Torrance, CA, USA).

### 2.10. Statistics

Statistical analysis was performed using GraphPad Prism, version 9.0 (La Jolla, CA, USA). The data were analyzed using one-way ANOVA with Šidák’s multiple comparisons test (* *p* < 0.05; ** *p* < 0.01; *** *p* < 0.001).

### 2.11. In Silico Methods

**Figure 1:**
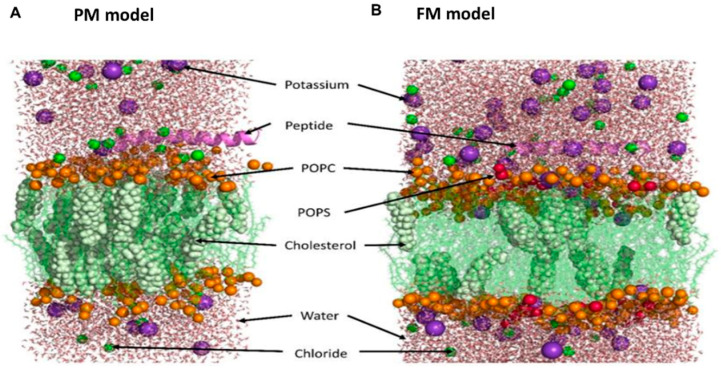
Models of peptide with (**A**) plasma membrane (PM) or (**B**) flavivirus membrane (FM) model used for simulations. Models are generated by CHARMM-GUI and visualized using PyMol. POPC:1-palmitoyl-2-oleoyl-sn-glycero3-phosphatidylcholine, POPS:1-palmitoyl-2-oleoyl-sn-glycero-3-phospho-L-serine.

Due to the lack of structural data for the peptides, AlphaFold [44] was employed to model the active sequences of PLNC8 α and PLNC8 β. Multiple systems were investigated using MD simulations, which included the active peptides PLNC8 α and PLNC8 β within lipid bilayer systems simulating membranes. These systems were solvated in water, and ions were added for charge neutralization. The assembly of all-atom molecular models for the peptides integrated into a flavivirus membrane (FM) and a plasma membrane (PM) mimicking models were built using the CHARMM-GUI server employing Membrane Builder/Multicomponent Assembler [45,46,47,48]. Controls included membrane models devoid of peptides. Both peptides were capped at the N- and C-terminus with NH_3_^+^ and COO-, respectively. The initial orientation of each peptide over the membrane was estimated using Orientations of Proteins in Membranes (OPM). The membrane normal was set at Z = 0, with the membrane normal presumed to be aligned parallel to the Z-axis. Each peptide was initially positioned by translating to 55 Å along the Z-axis. The flavivirus membrane was modelled using a combination of membrane lipids 1-palmitoyl-2-oleoyl-sn-glycero3-phosphatidylcholine (POPC), 1-palmitoyl-2-oleoyl-sn-glycero-3-phospho-L-serine (POPS), and cholesterol (Chol) in the ratio 81:11:8 [41]. For the PM model, a mix of POPC and Chol in a 70:30 ratio was used [41]. Details of various bilayer membrane systems used for MD simulations are shown in Table S1. A water thickness of 22.5 Å was set along both sides of the membrane leaflets, and the X-axis and Y-axis were adjusted to accommodate the peptides (PLNC8 α: 45 Å and PLNC8 β: 61 Å) within a hexagonal box. The peptide-membrane bilayer system was neutralized with potassium (K^+^) or chloride (Cl^−^) ions in a 0.15 M KCl aqueous solution. The generated lipid bilayer systems containing the peptides underwent energy minimization using the steepest descent method with constant temperature (310.15 K) via the v-rescale algorithm and pressure using Parrinello-Rahman. Each MD simulation was run in triplicate, totaling 1000 ns, using a time step of 2 fs. GROMACS (v 2022.2) [49] was used for all MD simulations, using the CHARMM36m forcefield [50]. In Figure 1, illustrations of the models of the PLNC8 peptides with both PM and FM are shown. The simulations were generated by CHARMM-GUI and visualized using PyMol [51].

The secondary structure of the peptides during simulation was analyzed using the *gmx do_dssp* (DSSP) [52]. Various parameters were derived from the simulation trajectories using GROMACS analysis tools [49]. The root mean-square fluctuation (RMSF) and root mean square deviation (RMSD) were computed with *gmx rmsf* and *gmx rms*, respectively. The radius of gyration (Rg) was determined using *gmx gyrate*. The number of hydrogen bonds between the peptides and membrane components (POPC, POPS, and Chol) was quantified with *gmx hbond*. Additionally, the distance of the center of mass (COM) of the peptide concerning the center of mass of lipids over time was evaluated with *gmx distance*. Membrane thickness and area per lipid were computed using LiPyphilic [53].

For the analysis of binding free energy and its decomposition, gmx_MMPBSA was used [54]. The binding free energy calculations were conducted for the final 50 ns, with a frame interval of 10 for all MD simulations. The binding energies between the peptide and the membrane components (POPC, POPS, and cholesterol) were computed at a 0.15 M salt concentration and a temperature of 310.15 K. In Poisson Boltzmann (PB) Decomposition Energies, the delta of Total Decomposition Contribution (TDC) was determined. An energy decomposition scheme was employed, which involved per-residue decomp with 1–4 EEL added to EEL and 1–4 VDW added to VDW potential terms for decomposition analysis.

## 3. Results

Development of novel and potent antiviral compounds is necessary, and it is advantageous if these compounds also can antagonize or modulate the effects of the viral infection on host cells, including cellular cytotoxicity and inflammation. In this study, we aimed to further investigate the antiviral properties of PLNC8 αβ and to explore how this peptide influences the cellular response to viral infections.

**Figure 2:**
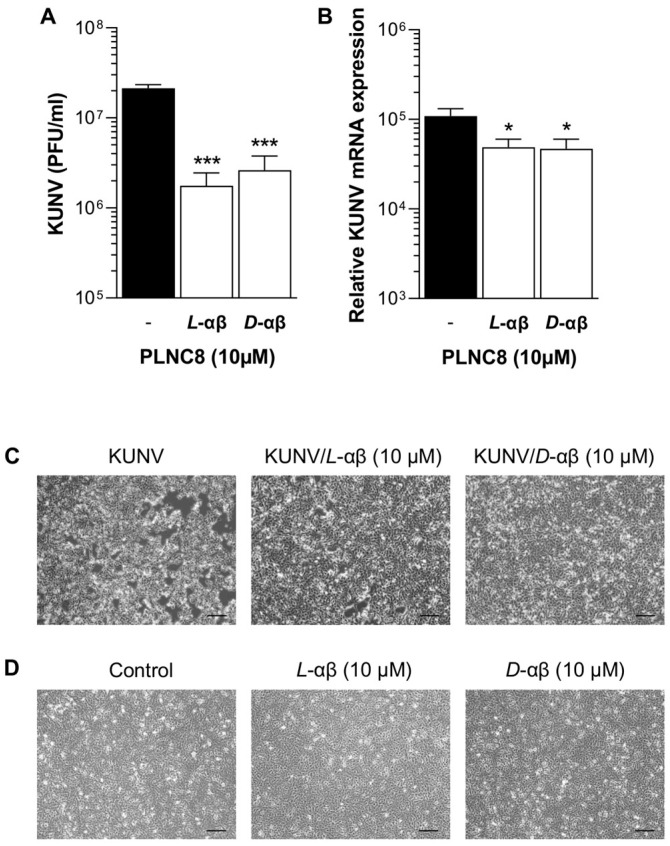
PLNC8 αβ targets extracellular viruses. Human lung carcinoma cells (A549) were infected with KUNV (MOI:0.1) for 1 h, followed by replacement of the cell culture media with fresh medium (DMEM supplemented with 2.5% FBS) containing *L*-PLNC8 αβ or *D*-PLNC8 αβ (*L*-αβ, *D*-αβ) at a final concentration of 10 µM. The cells were harvested after 48 h. (**A**) Extracellular virions (KUNV) were quantified in the cell culture media by plaque assay. (**B**) Viral replication was quantified by RT-PCR. (**C**) Representative images of human cells infected with KUNV, in the presence or absence of PLNC8 αβ, scale bar is 50 µm. (**D**) Representative images of human cells exposed to *L*-PLNC8 αβ or *D*-PLNC8 αβ at a final concentration of 10 µM, scale bar is 50 µm. One-way ANOVA with Šidák’s multiple comparisons test (* *p* < 0.05; *** *p* < 0.001).

The antiviral activity of PLNC8 αβ was investigated using human lung epithelial cells (A549) infected with KUNV. After an initial one-hour adsorption phase of KUNV at MOI of 0.1, new media was added, and the cells were incubated for 48 h in the presence or absence of 10 µM of either *L*-PLNC8 αβ or *D*-PLNC8 αβ. The number of released KUNV virions in untreated cells was substantially increased compared to the initial viral load and treatment with *L*-PLNC8 αβ or *D*-PLNC8 αβ caused a significant reduction of extracellular virions by >60% (Figure 2A). Analysis of intracellular viral RNA reflected the results of released extracellular virions, in which the peptides caused a significant reduction of KUNV mRNA levels compared to the untreated positive control (Figure 2B). Morphologically, KUNV-infected cells were markedly affected by appearing round, detached from the surface, and did not form a confluent layer. Treatment with *L*-PLNC8 αβ or *D*-PLNC8 αβ mitigated the detrimental effects of KUNV on cell morphology, as illustrated in Figure 2C. Exposure of human cells to the peptide alone did not affect cell viability (Figure 2D). The morphological changes induced by KUNV are primarily indicative of cell death, and the addition of PLNC8 αβ appears to preserve normal cellular morphology, most probably by limiting viral spread.

**Figure 3:**
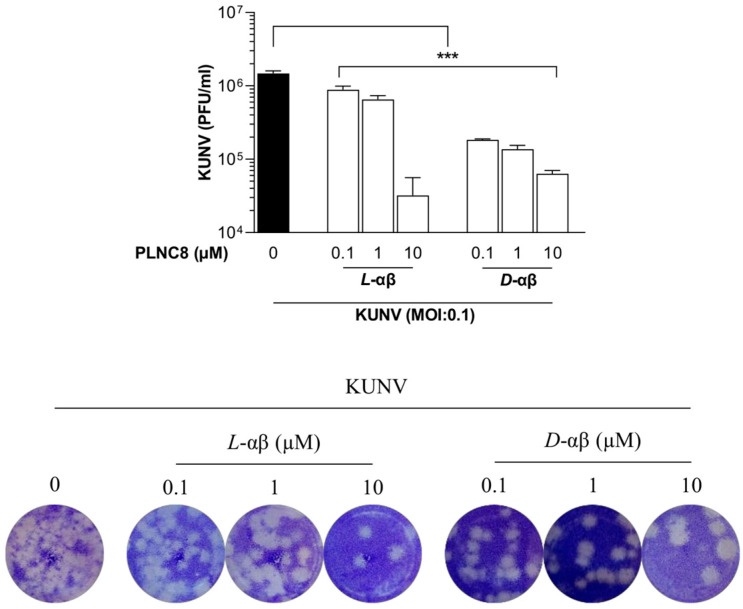
Significant reduction of the viral load after multiple administrations of PLNC8 αβ. Human lung carcinoma cells (A549) were infected with KUNV (MOI 0.1) for 1 h, followed by administration of *L*-PLNC8 αβ (*L*-αβ) or *D*-PLNC8 αβ (*D*-αβ) at 0.1, 1, or 10 µM once every 24 h for a total of three doses. Extracellular virions (KUNV) were quantified in the cell culture media by plaque assay. Representative images of crystal violet staining are also shown. One-way ANOVA with Šidák’s multiple comparisons test (*** *p* < 0.001).

To further explore the antiviral effects of PLNC8 αβ, we evaluated its activity in a dose-dependent manner and after administration of multiple-doses. Cells were infected with KUNV (MOI 0.1) and treated with three concentrations of either *L*-PLNC8 αβ or *D*-PLNC8 αβ (0.1, 1, and 10 µM), once every 24 h, and the viral load was quantified by plaque assay at 72 HPI. *L*-PLNC8 αβ at 0.1, 1, and 10 µM caused 40%, 60%, and >97% inhibition of extracellular virions, respectively, while the *D*-form of PLNC8 αβ was markedly more potent, causing 80–96% reduction of the number of infective virions, even at the lowest concentration of 0.1 µM (Figure 3).

**Figure 4:**
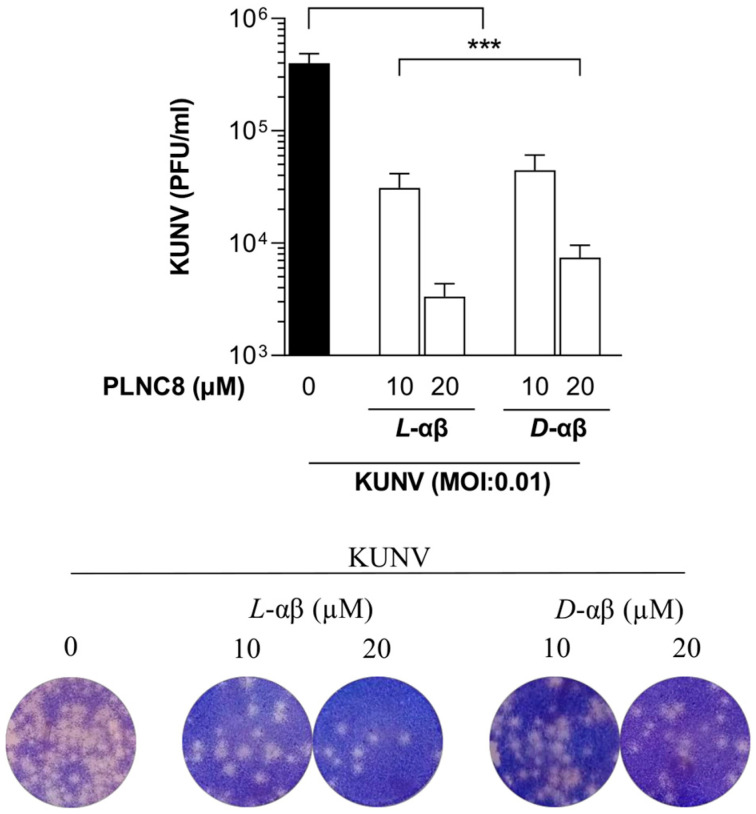
PLNC8 αβ significantly reduces the extracellular viral load of infected cells. Human lung carcinoma cells (A549) were infected with KUNV (MOI 0.01) for 24 h, followed by administration of *L*-PLNC8 αβ (*L*-αβ) or *D*-PLNC8 αβ (*D*-αβ) at 10 µM or 20 µM for 2 h. The viral load was determined by plaque assay and representative images of crystal violet staining are also shown. One-way ANOVA with Šidák’s multiple comparisons test (*** *p* < 0.001).

Furthermore, to confirm that PLNC8 αβ targets extracellular viruses, we investigated its antiviral activity when administered at a relatively late stage of virus infection (24 HPI). After an initial infection for 24 h, a final concentration of 10 or 20 µM of PLNC8 αβ caused a significant reduction in extracellular infective viral particles after 2 h of exposure to the peptides (Figure 4).

**Figure 5:**
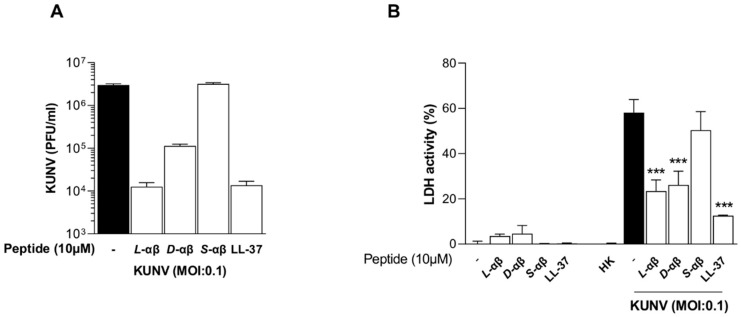
PLNC8 αβ reduces KUNV-induced cytotoxicity. Human lung carcinoma cells (A549) were infected with KUNV for 1 h, followed by treatment with 10 µM of *L*- or *D*-PLNC8 αβ (*L*-αβ, *D*-αβ) for 48 h. (**A**) Plaque assay of KUNV (PFU/mL). (**B**) Cytotoxicity was determined as relative LDH levels. Comparison of KUNV-infected cells with/without different treatment by using One-way ANOVA with Šidák’s multiple comparisons test (*** *p* < 0.001).

Given these findings, we examined the cytotoxicity induced by KUNV infection and the impact of PLNC8 αβ treatment. In these experiments we included additional controls; LL-37, scrambled PLNC8 αβ, and heat-inactivated viruses (Figure 5). Both *L*-PLNC8 αβ and *D*-PLNC8 αβ, as well as the human peptide LL-37, reduced the number of extracellular viruses significantly, while no antiviral activity was obtained using scrambled PLNC8 αβ (Figure 5A). Determination of extracellular LDH activity showed that the peptides are not cytotoxic, compared to the unexposed negative control (Figure 5B). Viable KUNV caused a significant amount of cell death after 48 h of infection, compared to heat-inactivated KUNV that did not cause any cytotoxic effects on the cells. This confirms that the cytotoxicity is due to inert virions and not inactivated viral particles or individual viral antigens. The presence of all peptide variants, except scrambled PLNC8 αβ, significantly suppressed the cytotoxic effects of KUNV.

**Figure 6:**
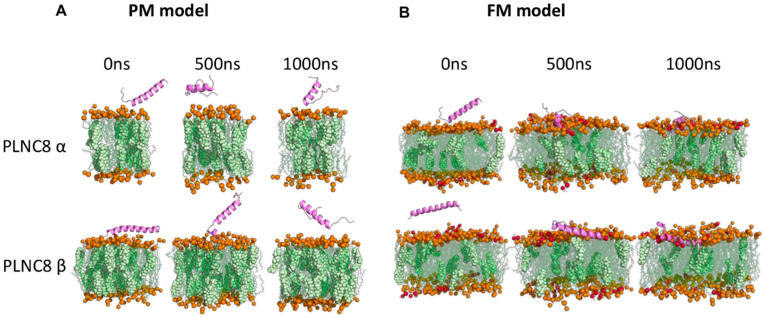
PLNC8 α and PLNC8 β show strong interactions with flavivirus membrane models. Representative illustration of PLNC8 α and PLNC8 β with (**A**) plasma membrane and (**B**) flavivirus membrane models at 0, 500 and 1000 ns. Lipid molecules POPC, POPS, cholesterol, and the peptide are depicted as dark yellow, red, purple, and off-white, respectively. Water and ions have been omitted for clarity.

The potent antiviral activity of PLNC8 αβ, compared with its low cytotoxic effects, suggests that the peptides preferentially target viral membranes. To confirm this, we conducted silico experiments using molecular dynamics (MD) simulation to investigate the interactions of PLNC8 α and PLNC8 β with flavivirus membrane (FM) and cellular plasma membrane (PM) models. The simulations revealed that both peptides exhibited a stronger propensity to bind and penetrate FM, which contain higher concentrations of negatively charged lipids (POPS), compared to the zwitterionic lipids (POPC) in PM (Figure 6).

Notably, PLNC8 β demonstrated superior binding efficacy to FM due to several positively charged residues contributing significantly to the interaction (Figure S1). In contrast, neither peptide showed significant interaction with the lipids in PM. Further analyses of peptide stability and structural dynamics revealed that PLNC8 β maintained its α-helical structure more effectively in both membrane types, especially in FM, and exhibited greater structural compactness and stability than PLNC8 α (Figures S2 and S3). Both peptides formed more hydrogen bonds with FM than with PM (Figure S4), though they did not induce significant changes in membrane thickness or lipid area (Figure S5). Lastly, by calculating the distance between the center of mass (COM) of the peptide and the COM of the lipids during the simulation run, it is obvious that both peptides penetrated FM more deeply than PM (Figure S6).

**Figure 7:**
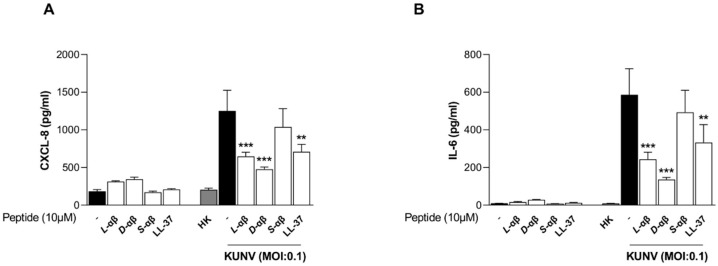
PLNC8 αβ alters the expression of inflammatory mediators. Human lung carcinoma cells (A549) were infected with KUNV for 1 h, followed by treatment with 10 µM of *L*- or *D*-PLNC8 αβ (*L*-αβ, *D*-αβ) for 48 h. The levels of inflammatory mediators (**A**) CXCL-8 and (**B**) IL-6, were determined in the cell culture media by ELISA. Comparison of KUNV-infected cells with/without different treatment by using One-way ANOVA with Šidák’s multiple comparisons test (** *p* < 0.01; *** *p* < 0.001).

Next, we assessed the impact of PLNC8 αβ on the inflammatory response during KUNV infection by measuring the secretion of CXCL-8 (Figure 7A) and IL-6 (Figure 7B). Exposure of cells to the peptides alone or to the heat-inactivated virus did not significantly alter the secretion of either cytokine, compared to the untreated negative control. In contrast, infection with inert KUNV triggered a marked inflammatory response, which was significantly suppressed by all peptide variants, except for scrambled PLNC8 αβ.

We further investigated the influence of PLNC8 αβ on inflammatory gene expression. We found minor effects of *L*-PLNC8 αβ or *D*-PLNC8 αβ alone in altering the transcriptional activity of cytokines in human lung epithelial cells. KUNV induced a severalfold increase in the transcription of *IL1β*, *IL6*, *TNF*, *CXCL8*, and *CCL5*, indicating establishment of an inflammatory state in the cells. Generally, *L*-PLNC8 αβ and *D*-PLNC8 αβ tend to counteract the KUNV-induced inflammatory gene expression of all cytokines, except for *IL6* and *CCL5* that were further upregulated in the presence of both enantiomers of PLNC8 αβ (Table 3).

We then examined intracellular signaling pathways activated by KUNV infection and PLNC8 αβ (Table 4). Analysis of key intracellular signaling molecules showed minor changes in PLNC8 αβ-exposed cells. Notably, the expression of the MAPK-activated *FOS* and *JUN* was significantly induced by KUNV and the presence of *L*-PLNC8 αβ or *D*-PLNC8 αβ markedly decreased this expression. In contrast, receptor activator of nuclear factor kappa Β ligand (RANKL) was significantly suppressed by KUNV, whereas treatment with the peptides enhanced its gene expression.

Furthermore, the levels of phosphorylated-p65 (p-p65) were determined in cell culture lysates from infected and non-infected cells, in the presence or absence of peptides. Both enantiomers of PLNC8 αβ caused a minor upregulation of p-p65, while KUNV caused a substantial downregulation of p-p65 (Figure S7). The levels of p-p65 were enhanced in KUNV-infected cells in the presence of PLNC8 αβ, compared to infected control cells. These results may indicate the involvement of the transcription factor NF-κB in response to KUNV infection and PLNC8 αβ treatment.

Finally, we explored the modulation of upstream host receptors by PLNC8 αβ. We show that exposure of cells to *D*-PLNC8 αβ, but not *L*-PLNC8 αβ, caused minor but significant suppression of *TLR1*, *TLR3*, *TLR6*, *PAR3*, and *NOD2*. KUNV infection altered the levels of almost all the investigated PRRs, including *TLR1*, *TLR6*, *PAR1*, *PAR3*, and *NOD1* that were significantly suppressed, while the expression of *TLR3*, *TLR9*, and *NOD2* were significantly upregulated. PLNC8 αβ counteracted the virus-induced expression of *TLR9* and *NOD2*, while *TLR3* levels were further induced by both enantiomers of PLNC8 αβ (Table 5).

## 4. Discussion

The role of antimicrobial peptides (AMPs) as potential agents against viral infections has increasingly been explored during recent years. Both direct antiviral activity (virucidal) and indirect effects (through modulation of inflammatory and immune responses) have been proposed as possible underlying mechanisms of action for these antiviral AMPs [16,30,42]. PLNC8 αβ is a promising AMP having both antibacterial and antiviral properties. We have previously reported its antibacterial activity against several bacteria [40,55]. Recently, we demonstrated a potent direct virucidal property of PLNC8 αβ against a wide range of enveloped viruses, particularly those which obtain their envelopes from the ER e.g., flaviviruses and SARS-CoV-2 [41].

The results of the current study clearly show that exposure of KUNV-infected cells to a single dose of either *L*-PLNC8 αβ or *D*-PLNC8 αβ significantly decreases both the extracellular and intracellular viral load. Furthermore, significant antiviral effects were also achieved with multiple doses of sub-micromolar concentration of the peptides, particularly the *D*-enantiomer of PLNC8 αβ. The direct virucidal activity of the peptides was determined by allowing the viral infection to proceed for 24 h followed by conducting a plaque assay immediately after a 2-h exposure time to the peptides. The significant reduction in the number of extracellular virions provided a clearer understanding of the direct antiviral mechanisms of PLNC8 αβ, confirming their effectiveness in targeting viral particles outside of host cells. Consequently, the reduction in intracellular viruses may be attributed to the decrease in the number of virions capable of infecting neighboring cells. Considering the antiviral activity of PLNC8 αβ, we explored the cytotoxic effects of KUNV infection and the potential protective role of PLNC8 αβ treatment. Both the *L*- and *D*-form of PLNC8 αβ demonstrated a significant reduction in virus-induced cytotoxicity, as evidenced by lower extracellular LDH levels. In contrast, scrambled PLNC8 αβ showed no antiviral activity, indicating the specificity of the protective effects by PLNC8 αβ against KUNV infection.

The potent antiviral activity of PLNC8 αβ against the flavivirus KUNV, coupled with its low cytotoxicity toward host cells, prompted us to investigate the molecular mechanisms driving this favorable selectivity. Understanding the specific interactions between PLNC8 αβ and viral versus cellular membranes is essential in order to understand the basis for its antiviral action and minimal impact on host cells. Certain viruses, such as flaviviruses, are known to acquire their envelopes from the endoplasmic reticulum (ER) membrane, which has a symmetric distribution of phospholipids across its inner and outer leaflets; whereas the plasma membrane (PM) is asymmetrical, with negatively charged phospholipids primarily localized on the cytosolic side. This asymmetry is maintained by both energy-dependent and energy-independent enzymes [56]. Additionally, the PM is more stable due to its higher cholesterol content [57]. Given these differences in electrochemical properties, we hypothesized that PLNC8 αβ, as a cationic peptide, selectively targets flaviviruses and other viruses with similar envelope origins, while sparing host cells. To test this, we employed in silico molecular dynamics simulations to model the interactions of PLNC8 α and PLNC8 β with phospholipid bilayers mimicking flavivirus membrane (FM) and cellular plasma membrane (PM). Our findings demonstrate that PLNC8 α and PLNC8 β exhibit stronger interactions and deeper penetration into the FM model compared to the PM model. This is evidenced by multiple parameters, including the retention of α-helicity, increased hydrogen bond formation, and enhanced structural stability and penetration depth during simulations. Notably, positively charged amino acid residues played a key role in peptide-lipid interactions, supporting our hypothesis that PLNC8 αβ preferentially interacts with the negatively charged phospholipids abundant in viral envelopes. These results align with previous observations using liposomal models of cellular membranes [41], reinforcing the idea that PLNC8 αβ exerts minimal cytotoxicity due to its limited interactions with eukaryotic cell PMs.

Given that many cationic peptides are known to modulate host inflammatory responses [58,59,60], we sought to investigate whether PLNC8 αβ exhibits similar immunomodulatory effects. We initially evaluated the effects of PLNC8 αβ on the expression of inflammatory cytokines, which is a common feature of the host response to viral infections and other pathogenic stimuli [61]. We have shown that PLNC8 αβ alters the expression and accumulation of certain cytokines in response to an infection by the flavivirus KUNV. Suppressed secretion of CXCL-8 and IL-6 is most likely dependent on the antiviral activity of PLNC8 αβ that reduces the cytotoxic effect of KUNV and its provocation of a stronger inflammatory response against the viruses. Furthermore, exposure of cells to PLNC8 αβ alone did not alter the expression of these cytokines, which may suggest that the observed effects on these inflammatory markers are secondary to the direct antiviral activity of the peptides. IL-6 is a pleiotropic cytokine with both proinflammatory and anti-inflammatory effects mediated through different signaling pathways [62]. Several studies have identified crucial roles for this cytokine in the effective host response to viral pathogens, such as influenza A virus (IAV) and Hepatitis B virus (HBV) [63,64]. In this study, however, we observed an increase in *IL6* gene expression, but a reduction in secreted IL-6 protein, in response to PLNC8 αβ treatment. This finding is noteworthy and indicates the involvement of intricate regulatory mechanisms, potentially including feedback inhibition or distinct regulation at the transcriptional and translational levels [65,66,67]. Antiviral activity mediated by TNF-α is a feature of host cell responses to many viruses, including WNV [68]. However, other studies suggest a pathologic role of this cytokine in the context of respiratory viral diseases, such as infections caused by IAV [69]. Although PLNC8 αβ significantly suppressed the expression of *TNF* in response to KUNV infection, the levels were generally low. Furthermore, PLNC8 αβ induced the gene expression of *CCL5* (also known as RANTES), a chemokine that plays important roles in the control of respiratory viral pathogenesis [70,71]. Recently, Silva et al. characterized a critical role of CCL-5 in the inhibition of IAV by the respiratory cell line A549 cells [72].

Investigating the effects of KUNV and PLNC8 αβ on key intracellular signaling pathways, such as the NF-κB and MAPK, is important to understand the regulatory mechanisms behind the altered levels of cytokine and chemokine expression. Interestingly, *FOS* and *JUN* were significantly induced by KUNV, indicating a role for the transcription factor activator protein (AP)-1 in regulating inflammatory gene expression. These effects were counteracted by PLNC8 αβ, particularly the *D*-form which suppressed *FOS* expression to basal levels. C-Fos and c-Jun belong to the AP-1 group of transcriptional factors that have been linked to several critical cellular processes, such as inflammation, proliferation, differentiation, and apoptosis [73]. AP-1 is known to be activated by members of the MAPK superfamily, such as JNKs [74]. Previous studies have indicated that AP-1 is stimulated in response to several viral pathogens, such IAV, Human Immunodeficiency Virus type1 (HIV-1) and Herpes Simplex Virus type2 (HSV-2) [75,76,77]. In line with this, our results showed higher levels of phosphorylated p65 in the PLNC8 αβ-treated cells, indicating the involvement of NF-κB in the initiation of an inflammatory response and cell survival. The role of NF-κB in cellular processes, including cell survival, proliferation, and inflammation is well documented [26,78,79].

Viruses are intracellular pathogens that are mainly detected by endosomal TLRs, such as TLR3, 7, 8, and 9 [80]. In this study, we show that PLNC8 αβ significantly enhanced cellular gene expression of *TLR3* in response to KUNV infection, while *TLR9* expression was substantially suppressed. The effect on *TLR3* is particularly interesting given its well-characterized role in sensing double-stranded RNA and stimulating strong antiviral and inflammatory pathways [81]. Moreover, a role of TLR3 in the protection against the flavivirus WNV has previously been demonstrated, in which activation of TLR3 restricted the replication of the virus, possibly by enhancing rapid sensing of viral RNA and by generating effective interferon response [18]. On the other hand, our results show that *TLR9* is induced by KUNV and that PLNC8 αβ counteracts this effect. This is interesting because KUNV is an RNA virus and TLR9 is mostly involved in the recognition of unmethylated CpG motifs in DNA viruses, such as cytomegalovirus (CMV) and HSV-2 [82,83]. However, emerging evidence suggests that other factors, such as mitochondrial DNA leakage, can also trigger TLR9 signaling pathways in the context of viral infections caused by RNA viruses, such as the flavivirus DENV and SARS-CoV-2 [84,85]. Therefore, it is plausible that KUNV may also induce similar mitochondrial perturbations leading to the observed induction of *TLR9*. We also evaluated the effects KUNV and PLNC8 αβ on *NOD1* and *NOD2* expression. These receptors belong to the NLRs group of cytosolic PRRs that are involved in the cellular response to intracellular pathogens [86]. PLNC8 αβ significantly suppresses the virally induced expression of *NOD2*. This is particularly interesting in view of recent studies indicating a pathogenic role of this molecule in the context of flavivirus (ZIKA) infection and that drugs blocking NOD-2 significantly suppressed the replication of a wide range of viruses, including flaviviruses and SARS-CoV-2 [87]. Furthermore, PLNC8 αβ induced the expression of *NOD1*, which has previously been shown to enhance the antiviral mechanisms by restricting the replication of viral RNA [88]. Protease-activated receptors (PARs) do not appear to play a major role in KUNV infection, and were not altered by the peptides, except for *PAR1* that was suppressed by KUNV and significantly induced following an addition of *D*-PLNC8 αβ. Important roles of PARs are well documented in the innate immune response to bacterial infections, and some studies also indicate a possible protective function of PARs against viral infection, such as IAV [89,90].

In conclusion, our study demonstrates the potent antiviral activity of PLNC8 αβ against the flavivirus KUNV, alongside its previously reported efficacy against other enveloped viruses like SARS-CoV-2. The findings suggest that the antiviral mechanism of PLNC8 αβ is primarily due to its ability to selectively target and disrupt viral membranes, without displaying cytotoxic effects on host cells. This selectivity appears to stem from the preferential interaction of the peptides with the negatively charged phospholipids abundant in the flavivirus membranes, as supported by molecular dynamics simulations. Furthermore, PLNC8 αβ modulates KUNV-induced host inflammatory responses, particularly by altering the expression of key cytokines and signaling pathways. Overall, the findings of this study reinforce the potential of PLNC8 αβ as a promising candidate in the development of novel antiviral therapies.

## Figures and Tables

**Table 1:** Amino acid sequence, molecular weight, and net charge at pH 7 of the peptide *L*/*D*/*S*-PLNC8 α, *L*/*D*/*S*-PLNC8 β and LL-37 that were used in this study. LL-37 is a human Cathelicidin antimicrobial peptide and was used as a control in this study as it has previously been shown to display antiviral properties against several viruses, including some flaviviruses [32,42,43].

Name	Sequence	MW	Net Charge at pH 7
*L*-PLNC8 α	DLTTKLWSSWGYYLGKKARWNLKHPYVQF	3587	4.1
*D*-PLNC8 α	DLTTKLWSSWGYYLGKKARWNLKHPYVQF	3587	4.1
*S*-PLNC8 α	TWLKYGHGDAKLWSWSKPLNLTFRYQYRK	3587	4.1
*L*-PLNC8 β	SVPTSVYTLGIKILWSAYKHRKTIEKSFNKGFYH	4001	5.2
*D*-PLNC8 β	SVPTSVYTLGIKILWSAYKHRKTIEKSFNKGFYH	4001	5.2
*S*-PLNC8 β	LKLWNTYGTFSRFYTSKSEVKIAHGIKSIHVPYK	4001	5.2
LL-37	LLGDFFRKSKEKIGKEFKRIVQRIKDFLRNLVPRTES	4493	6.0

**Table 2:** Primer sequences that were used to analyze gene expression patterns in human cells that were infected with KUNV, in the presence or absence of *L*-PLNC8 αβ or *D*-PLNC8 αβ.

Gene	Oligo	Primer Sequence
*GAPDH*	Forward	GTCTCCTCTGACTTCAACAGCG
	Reverse	ACCACCCTGTTGCTGTAGCCAA
*KUNV*	Forward	CACCACTACAGAGAGTGGAAAG
	Reverse	ATCATGTCTCTGTGGCCTAATC
*TLR1*	Forward	CAGCGATGTGTTCGGTTTTCCG
	Reverse	GATGGGCAAAGCATGTGGACCA
*TLR2*	Forward	CTTCACTCAGGAGCAGCAAGCA
	Reverse	ACACCAGTGCTGTCCTGTGACA
*TLR3*	Forward	GCGCTAAAAAGTGAAGAACTGGAT
	Reverse	GCTGGACATTGTTCAGAAAGAGG
*TLR4*	Forward	CCCTGAGGCATTTAGGCAGCTA
	Reverse	AGGTAGAGAGGTGGCTTAGGCT
*TLR6*	Forward	ACTGACCTTCCTGGATGTGGCA
	Reverse	TGACCTCATCTTCTGGCAGCTC
*TLR9*	Forward	TGAGCCACAACTGCATCTCGCA
	Reverse	CAGTCGTGGTAGCTCCGTGAAT
*PAR1*	Forward	GTTTCTGGCTGTGGTGTATCCC
	Reverse	CCTGGATGGTTTGCTCCTTGAG
*PAR2*	Forward	CTCCTCTCTGTCATCTGGTTCC
	Reverse	TGCACACTGAGGCAGGTCATGA
*PAR3*	Forward	CTTGGCAAAGCCAACCTTACCC
	Reverse	CAGTAATCGTGGCTCCTGTCCA
*NOD1*	Forward	CAACGGCATCTCCACAGAAGGA
	Reverse	CCAAACTCTCTGCCACTTCATCG
*NOD2*	Forward	GCACTGATGCTGGCAAAGAACG
	Reverse	CTTCAGTCCTTCTGCGAGAGAAC
*RANKL*	Forward	GCCTTTCAAGGAGCTGTGCAAAA
	Reverse	GAGCAAAAGGCTGAGCTTGAAGC
*RELA*	Forward	TGAACCGAAACTCTGGCAGCTG
	Reverse	CATCAGCTTGCGAAAAGGAGCC
*JUN*	Forward	CCTTGAAAGCTCAGAACTCGGAG
	Reverse	TGCTGCGTTAGCATGAGTTGGC
*FOS*	Forward	GCCTCTCTTACTACCACTCACC
	Reverse	AGATGGCAGTGACCGTGGGAAT
*STAT5*	Forward	GTTCAGTGTTGGCAGCAATGAGC
	Reverse	AGCACAGTAGCCGTGGCATTGT
*IL1B*	Forward	CCACAGACCTTCCAGGAGAATG
	Reverse	GTGCAGTTCAGTGATCGTACAGG
*CXCL8*	Forward	GAGAGTGATTGAGAGTGGACCAC
	Reverse	CACAACCCTCTGCACCCAGTTT
*IL6*	Forward	AGACAGCCACTCACCTCTTCAG
	Reverse	TTCTGCCAGTGCCTCTTTGCTG
*TNF*	Forward	CTCTTCTGCCTGCTGCACTTTG
	Reverse	ATGGGCTACAGGCTTGTCACTC
*CCL5*	Forward	CCTGCTGCTTTGCCTACATTGC
	Reverse	ACACACTTGGCGGTTCTTTCGG
*IFNA*	Forward	AGCCATCTCTGTCCTCCATGA
	Reverse	CATGATTTCTGCTCTGACAACC
*TNFB1*	Forward	TACCTGAACCCGTGTTGCTCTC
	Reverse	GTTGCTGAGGTATCGCCAGGAA

**Table 3:** Gene expression analysis of cytokines and chemokines. Human lung carcinoma cells (A549) were infected with KUNV for 1 h, followed by treatment with 10 µM of *L*-PLNC8 αβ (*L*-αβ) or *D*-PLNC8 αβ (*D*-αβ) for 48 h. Gene expression was analyzed with RT-PCR. The data (n = 3) are presented as mean with SD and statistics, at one significance level (*p* < 0.05), were analyzed using one-way ANOVA with Šidák’s multiple comparison test (n = 3, significance compared to the * unexposed negative control and ^#^ KUNV-infected control).

	Un-Infected		KUNV (MOI:0.1)		
Gene	*L*-αβ	*D*-αβ	-	*L*-αβ	*D*-αβ
*IL1B*	1.9 ± 1.0 *	1.7 ± 0.8 *	10.2 ± 3.6 *	5.1 ± 3.2 *^#^	1.7 ± 0.7 *^#^
*IL6*	1.3 ± 1.7	2.8 ± 1.0 *	120.4 ± 42.2 *	556.9 ± 295.1 *^#^	276.1 ± 165.6 *
*TNF*	0.8 ± 0.6	1.5 ± 2.9	90.4 ± 73.4	36.7 ± 30.2	6.5 ± 4.5
*CXCL8*	0.8 ± 0.6	0.9 ± 0.6	68.5 ± 11.3 *	24.9 ± 17.1 *^#^	7.3 ± 8.1 *^#^
*CCL5*	0.9 ± 0.2	0.9 ± 0.2	7.1 ± 3.2 *	25.5 ± 11.9 *^#^	31.3 ± 8.8 *^#^
*IFNA*	3.0 ± 3.8	2.7 ± 1.9	8.2 ± 5.6	4.5 ± 3.2	0.6 ± 0.4
*TGFB1*	1.8 ± 1.9	1.7 ± 1.2	1.4 ± 1.6	2.0 ± 1.9	2.1 ± 1.9

**Table 4:** Gene expression analysis of intracellular signaling molecules. Human lung carcinoma cells (A549) were infected with KUNV for 1 h, followed by treatment with 10 µM of *L*- or *D*-PLNC8 αβ (*L*-αβ, *D*-αβ). (A) Gene expression levels of intracellular signaling molecules were analyzed with RT-PCR. The data (n = 3) are presented as mean with SD and statistics, at one significance level (*p* < 0.05), were analyzed using one-way ANOVA with Šidák’s multiple comparison test (n = 3, significance compared to the * unexposed negative control and ^#^ KUNV-infected control).

	Un-INFECTED		KUNV (MOI:0.1)		
Gene	*L*-αβ	*D*-αβ	-	*L*-αβ	*D*-αβ
*RANKL*	1.0 ± 0.6	1.9 ± 0.9 *	0.7 ± 0.2 *	4.2 ± 2.0 *^#^	2.1 ± 1.1 ^#^
*RELA*	0.8 ± 0.5	0.9 ± 0.5	1.7 ± 0.9	2.2 ± 1.4 *	1.3 ± 0.8
*FOS*	0.6 ± 0.4 *	0.9 ± 0.5	19.9 ± 8.6 *	4.9 ± 3.2 *^#^	0.9 ± 0.4 ^#^
*JUN*	0.7 ± 0.3 *	1.3 ± 0.4	9.5 ± 2.9 *	3.4 ± 1.6 *^#^	2.5 ± 1.0 *^#^
*STAT1*	1.0 ± 0.3	0.8 ± 0.5	2.9 ± 1.6	2.6 ± 1.1	2.3 ± 0.8 *

**Table 5:** Receptor gene expression analysis. Human lung carcinoma cells (A549) were infected with KUNV for 1 h, followed by treatment with 10 µM of *L*- or *D*-PLNC8 αβ (*L*-αβ, *D*-αβ) for 48 h. Gene expression was analyzed with RT-PCR. The data (n = 3) are presented as mean with SD and statistics, at one significance level (*p* < 0.05), were analyzed using one-way ANOVA with Šidák’s multiple comparison test (n = 3, significance compared to the * unexposed negative control and ^#^ KUNV-infected control).

	Un-Infected		KUNV (MOI:0.1)		
Gene	*L*-αβ	*D*-αβ	-	*L*-αβ	*D*-αβ
*TLR1*	1.2 ± 0.6	0.2 ± 0.1 *	0.4 ± 0.2 *	0.9 ± 0.5 ^#^	0.6 ± 0.2 *
*TLR3*	0.9 ± 0.5	0.4 ± 0.1 *	9.5 ± 3.3 *	26.7 ± 4.7 *^#^	18.3 ± 5.9 *^#^
*TLR6*	1.2 ± 0.5	0.4 ± 0.2 *	0.4 ± 0.2 *	0.8 ± 0.4 ^#^	0.7 ± 0.2 *^#^
*TLR9*	2.9 ± 1.8	3.1 ± 2.1	11.3 ± 6.6 *	0.9 ± 0.6 ^#^	3.6 ± 1.7 *^#^
*PAR1*	0.8 ± 0.3	1.2 ± 0.4	0.4 ± 0.3 *	0.6 ± 0.4 *	2.2 ± 1.7 ^#^
*PAR2*	1.3 ± 0.5	1.5 ± 0.7	1.6 ± 0.7	1.1 ± 0.4	0.9 ± 0.3
*PAR3*	0.5 ± 0.2 *	0.4 ± 0.1 *	0.2 ± 0.2 *	0.4 ± 0.5 *	0.5 ± 0.5
*NOD1*	0.9 ± 0.6	1.0 ± 0.7	0.7 ± 0.2 *	1.5 ± 0.4 *^#^	2.1 ± 0.5 *^#^
*NOD2*	0.9 ± 0.3	0.2 ± 0.1 *	88.1 ± 37.5 *	27.7 ± 8.0 *^#^	22.8 ± 6.7 *^#^

## Data Availability

The original contributions presented in the study are included in the article/Supplementary Materials; further inquiries can be directed to the corresponding author.

## Supplementary Materials

The following supporting information can be downloaded at: https://www.mdpi.com/article/10.3390/v16111770/s1, Table S1: Details of various bilayer membrane systems used for MD simulations; Figure S1: Binding free energy analysis; Figure S2: Secondary structure analysis of PLNC8 α and PLNC8 β peptides; Figure S3: DSSP α-helicity, RMSD, RMSF and Rg; Figure S4: Hydrogen bond analysis; Figure S5: Membrane thickness and area per lipid (APL); Figure S6: Distance of center of mass (COM) of the peptide from the COM of the lipids; Figure S7: P-p65 levels.
