# Supplementary material for: PLNC8 αβ Potently Inhibits the Flavivirus Kunjin and Modulates Inflammatory and Intracellular Signaling Responses of Alveolar Epithelial Cells
Source: Viruses. 2024 Nov 13;16(11):1770. doi: 10.3390/v16111770 (PMC11599086; doi:10.3390/v16111770)
Supplement: Supplementary file 1 [file viruses-16-01770-s001.zip › viruses-3293234-supplementary.pdf]

Table S1

System	Time (in ns)	Total No. of lipids[1]	Each lipid	Total No. of atoms	Mimicking system ratio
**PM PLNC8 α**	1000 × 3	30	POPC: 21, CHL:09	18547	Plasma membrane (POPC:CHL - 70:30)
**PM PLNC8 β**	1000 × 3	60	POPC: 42, CHL:18	37131	Plasma membrane (POPC:CHL - 70:30)
**PM Membrane Only**	1000 × 3	60	POPC: 42, CHL:18	28326	Plasma membrane (POPC:CHL - 70:30)
**FM PLNC8 α**	1000 × 3	100	POPC: 81, POPS: 11, CHL:08	67404	Flaviviral membrane (POPC:POPS:CHL - 81:11:08)
**FM PLNC8 β**	1000 × 3	100	POPC: 81, POPS: 11, CHL:08	67378	Flaviviral membrane (POPC:POPS:CHL - 81:11:08)
**FM Membrane Only**	1000 × 3	100	POPC: 81, POPS: 11, CHL:08	51577	Flaviviral membrane (POPC:POPS:CHL - 81:11:08)

[1] Total number of lipids on each leaflet.

Table S1: Details of various bilayer membrane systems used for MD simulations. Mimicking system for the plasma membrane (PM) and the flavivirus membrane (FM) models is adopted from [1]. The FM membranes were modelled using a combination of membrane lipids 1-palmitoyl-2-oleoyl-sn-glycero3-phosphatidylcholine (POPC), 1-palmitoyl-2-oleoyl-sn-glycero-3-phospho-L-serine (POPS), and cholesterol (Chol) in the ratio 81:11:8. For the PM model, a mix of POPC and Chol in a 70:30 ratio was used.

Figure S1

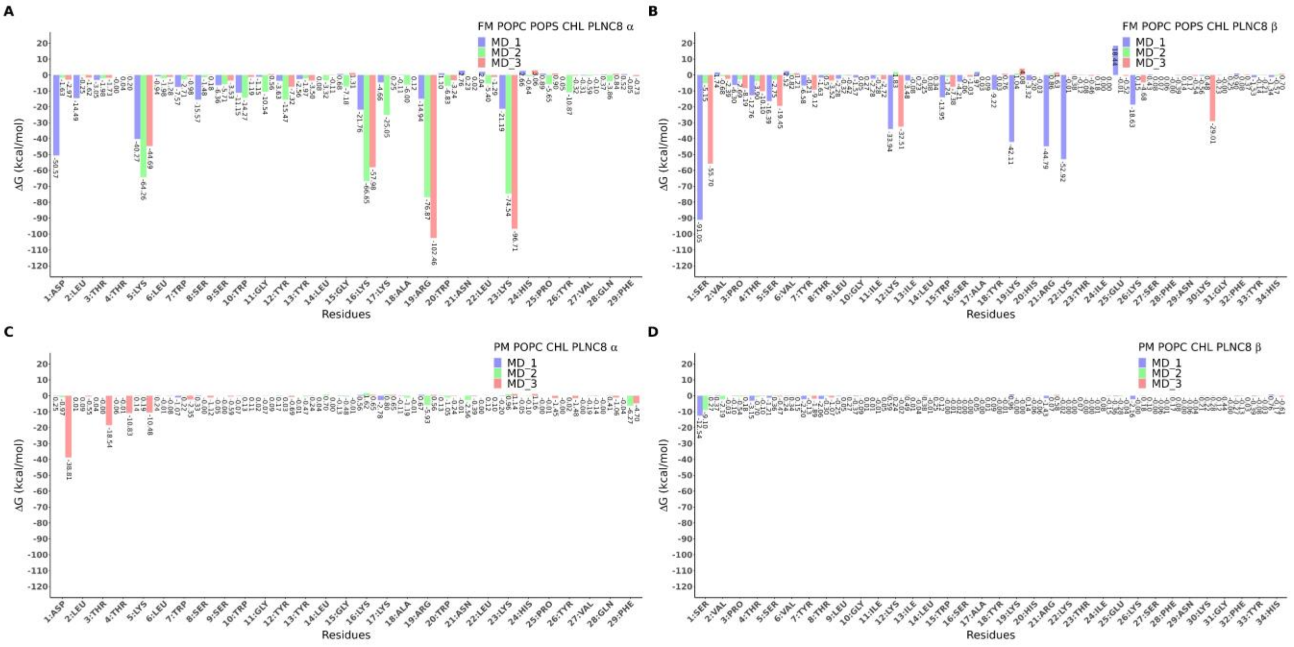

Figure S1: Binding free energy analysis (gmx_MMPBSA) of (**A**) PLNC8 α and (**B**) PLNC8 β in the flavivirus membrane (FM) model, (**C**) PLNC8 α and (**D**) PLNC8 β in the plasma membrane (PM) model. Different colours represent binding free energy from three independent simulations.

Figure S2

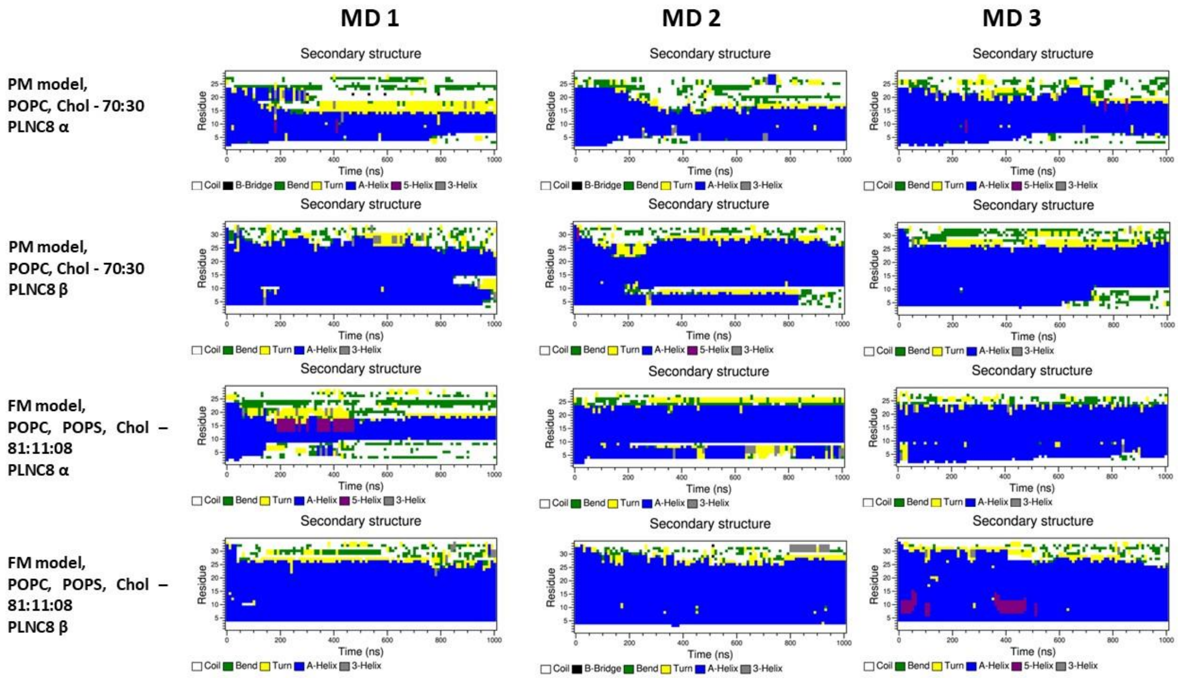

Figure S2. Secondary Structure Analysis of PLNC8 α and PLNC8 β Peptides. The secondary structure analysis of PLNC8 α and PLNC8 β peptides during a 1000 ns simulation in flavivirus membrane (FM) and plasma membrane (PM) models was performed using the *gmx do_dssp* tool. The simulations are denoted as MD_1, MD_2, and MD_3, signifying three separate and independent simulation runs.

Figure S3

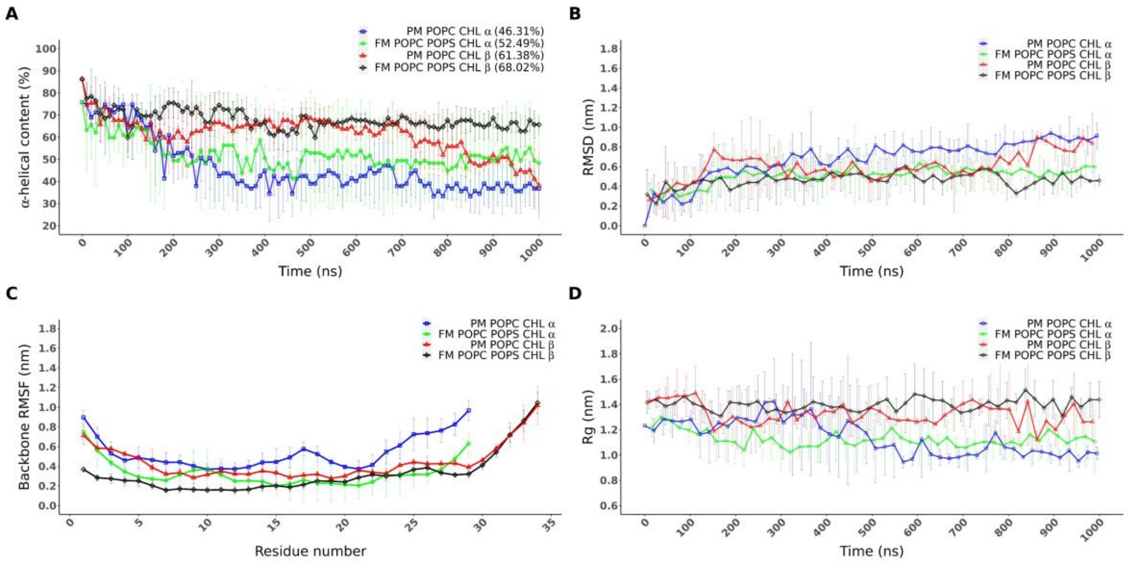

Figure S3. **DSSP α-helicity, RMSD, RMSF and Rg**. (**A**) Analysis of α-helicity using DSSP for peptides PLNC8 α and PLNC8 β in flavivirus membrane (FM) and plasma membrane (PM) models. The values in parentheses represent the average values throughout the entire duration of the MD simulation. (**B**) RMSD of PLNC8 α and PLNC8 β in FM and PM membrane models. (**C**) RMSF of PLNC8 α and PLNC8 β in FM and PM membrane models. (**D**) Radius of gyration (Rg) of PLNC8 α and PLNC8 β in FM and PM membrane models. The solid line represents the average value, and the shaded error bar depicts standard deviation from three independent simulation replicates.

Figure S4

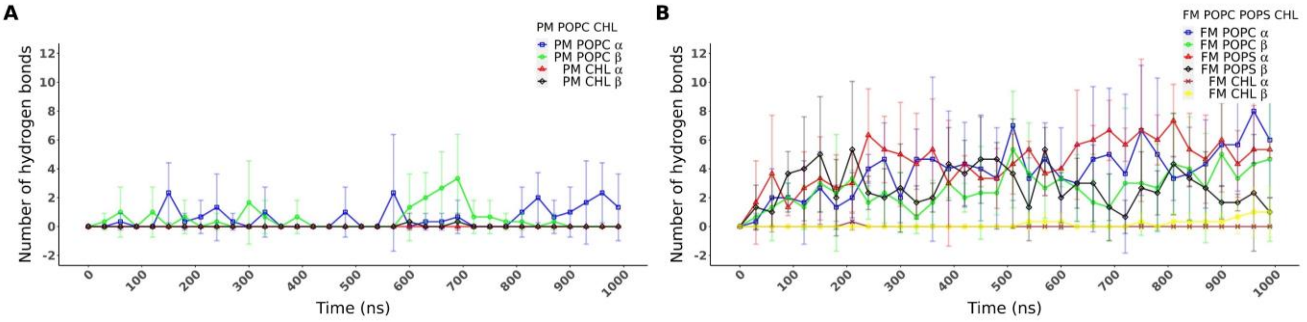

Figure S4. Hydrogen Bond Analysis. Number of hydrogen bonds between peptides and lipids (POPC, POPS, and Cholesterol) during 1000 ns simulation. (**A**) Hydrogen bonds formed between peptides PLNC8 α and PLNC8 β in the plasma membrane (PM) model. (**B**) Hydrogen bonds formed between peptides PLNC8 α and PLNC8 β in the flavivirus membrane (FM) model. The solid line represents the average value, and the shaded error bar depicts standard deviation from three independent simulation replicates.

Figure S5

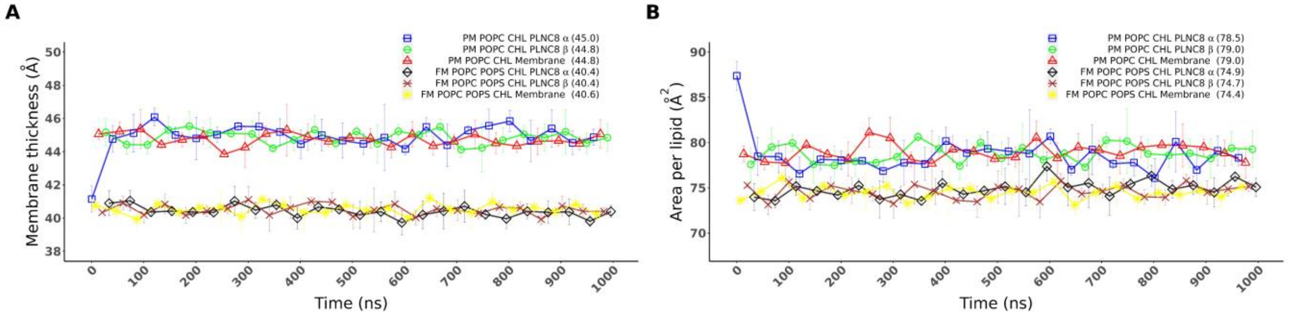

Figure S5. Membrane thickness and area per lipid (APL). (**A**) Alterations in membrane thickness for flaviviral membrane (FM) and plasma membrane (PM) models in the presence of PLNC8 α and PLNC8 β. (**B**) Changes in area per lipid (APL) due to the presence of PLNC8 α and PLNC8 β in PM and FM membrane models. The values in parentheses represent the average values throughout the entire duration of the MD simulation. The solid line represents the average value, and the shaded error bar depicts standard deviation from three independent simulation replicates.

Figure S6

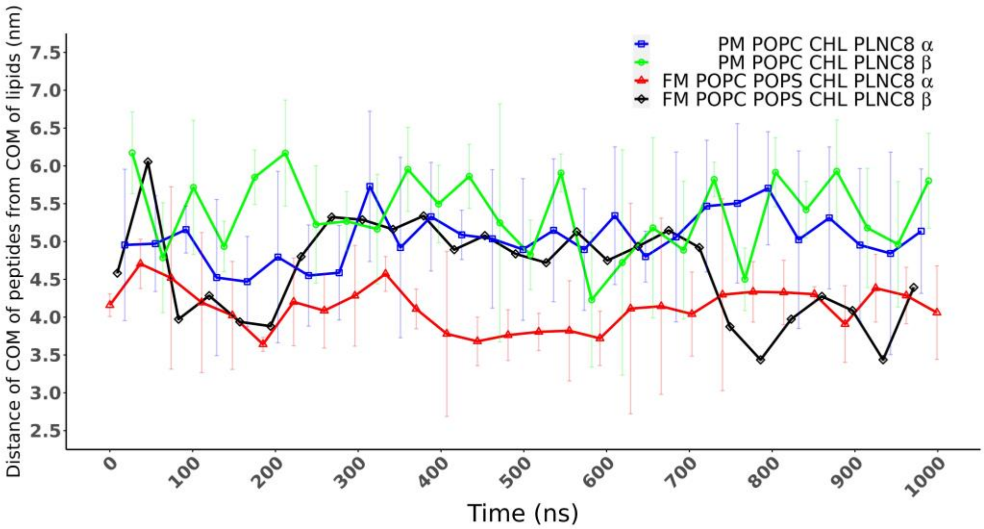

Figure S6. Distance of centre of mass (COM) of the peptide from the COM of the lipids: The solid line represents the average value, and the shaded error bar depicts standard deviation from three independent simulation replicates

Figure S7

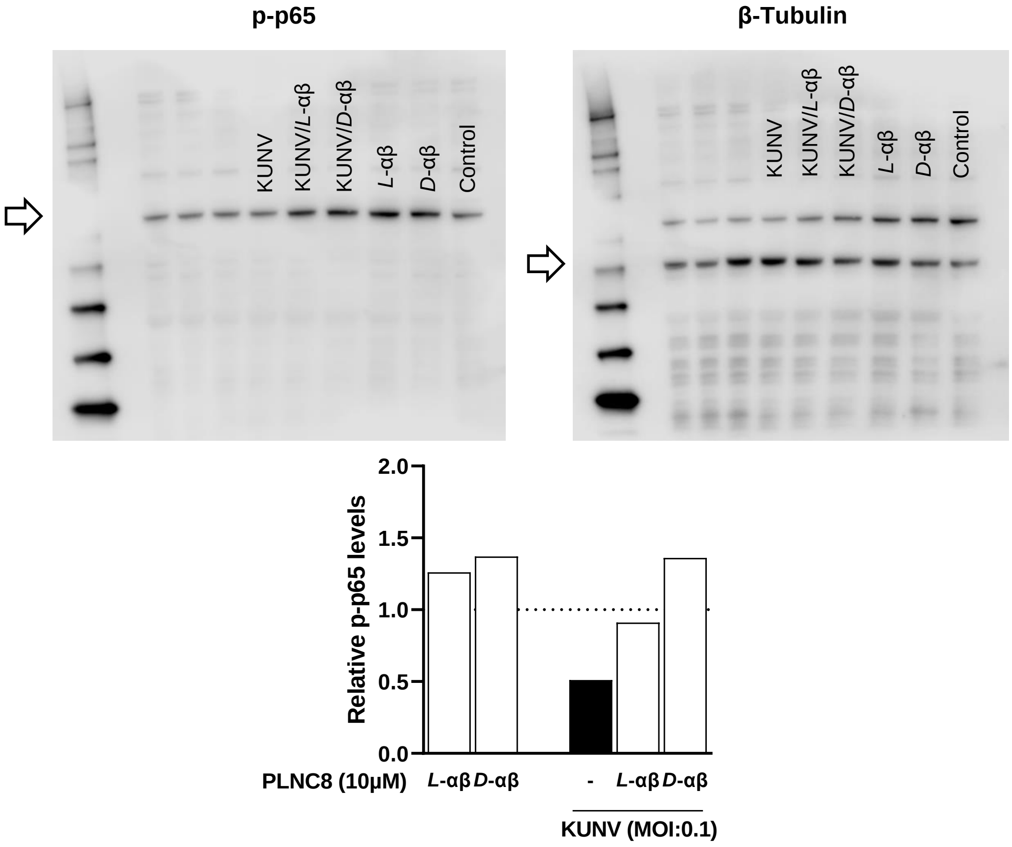

Figure S7. p-p65 levels in the different treatments were analyzed in cell culture lysates after infection with KUNV for 1 h and treatment with the peptides for 24 h. The blot is shown together with relative quantification of the band intensity that was normalized against β-tubulin and to the untreated negative control.
